# Sleep disorders and cancer incidence: examining duration and severity of diagnosis among veterans

**DOI:** 10.3389/fonc.2024.1336487

**Published:** 2024-02-26

**Authors:** James B. Burch, Alexandria F. Delage, Hongmei Zhang, Alexander C. McLain, Meredith A. Ray, Austin Miller, Swann A. Adams, James R. Hébert

**Affiliations:** ^1^ Department of Epidemiology, School of Population Health, Virginia Commonwealth University, Richmond, VA, United States; ^2^ Department of Epidemiology and Biostatistics, Arnold School of Public Health, University of South Carolina, Columbia, SC, United States; ^3^ Palmetto GBA, Columbia, SC, United States; ^4^ Division of Epidemiology, Biostatistics, and Environmental Health, School of Public Health, University of Memphis, Memphis, TN, United States; ^5^ Alabama College of Osteopathic Medicine, Dothan, AL, United States; ^6^ Department of Biobehavioral Health and Nursing Science, College of Nursing, University of South Carolina, Columbia, SC, United States; ^7^ South Carolina Statewide Cancer Prevention & Control Program, University of South Carolina, Columbia, SC, United States

**Keywords:** apnea, cancer, insomnia, risk, sleep, veteran

## Abstract

**Introduction:**

Sleep disruption affects biological processes that facilitate carcinogenesis. This retrospective cohort study used de-identified data from the Veterans Administration (VA) electronic medical record system to test the hypothesis that patients with diagnosed sleep disorders had an increased risk of prostate, breast, colorectal, or other cancers (1999-2010, N=663,869). This study builds upon existing evidence by examining whether patients with more severe or longer-duration diagnoses were at a greater risk of these cancers relative to those with a less severe or shorter duration sleep disorder.

**Methods:**

Incident cancer cases were identified in the VA Tumor Registry and sleep disorders were defined by International Classification of Sleep Disorder codes. Analyses were performed using extended Cox regression with sleep disorder diagnosis as a time-varying covariate.

**Results:**

Sleep disorders were present among 56,055 eligible patients (8% of the study population); sleep apnea (46%) and insomnia (40%) were the most common diagnoses. There were 18,181 cancer diagnoses (41% prostate, 12% colorectal, 1% female breast, 46% other). The hazard ratio (HR) for a cancer diagnosis was 1.45 (95% confidence interval [CI]: 1.37, 1.54) among those with any sleep disorder, after adjustment for age, sex, state of residence, and marital status. Risks increased with increasing sleep disorder duration (short [<1-2 years] HR: 1.04 [CI: 1.03-1.06], medium [>2-5 years] 1.23 [1.16-1.32]; long [>5-12 years] 1.52 [1.34-1.73]). Risks also increased with increasing sleep disorder severity using cumulative sleep disorder treatments as a surrogate exposure; African Americans with more severe disorders had greater risks relative to those with fewer treatments and other race groups. Results among patients with only sleep apnea, insomnia, or another sleep disorder were similar to those for all sleep disorders combined.

**Discussion:**

The findings are consistent with other studies indicating that sleep disruption is a cancer risk factor. Optimal sleep and appropriate sleep disorder management are modifiable risk factors that may facilitate cancer prevention.

## Introduction

Sleep disorders arise due to complex circumstances that can include genetic, environmental, behavioral, and psychosocial risk factors (1, 2). Veterans comprise a vulnerable population for sleep disorder occurrence and their rates are elevated relative to the general population (3, 4). Furthermore, secular trends in sleep disorder diagnoses have been increasing, both among Veterans and within the general population (3–5). For example, a national study of sleep disorder diagnoses among Veterans from 2000 to 2010 found a ~6-fold increase in sleep disorder prevalence during that period, with the largest increases among those with combat experience or post-traumatic stress disorder (3). Because of these increasing trends, an association between sleep disorders and cancer incidence would have important public health implications.

Several pathophysiological processes may mediate the association between chronic sleep disruption and carcinogenesis, including pathological changes in immune (via inflammation- and oxidative stress-related mechanisms), endocrine, neurological, metabolic and circadian systems (6–9). For example, the intermittent hypoxia and subsequent oxidative stress that occurs with sleep apnea has been cited as a biologically plausible, experimentally supported mechanism (7, 9). Results from longitudinal studies of sleep disorders and cancer incidence tend to show increased risks although results have been inconsistent. For example, several investigators reported no association between insomnia symptoms and prostate cancer (PrCA) incidence (10–12), whereas others report higher risks for PrCA among insomniacs after ~10 or more years of follow-up (13). Insomnia has also been associated with increased risks for breast (BrCA), colorectal (CRC) or other cancer types, particularly after a decade or more of follow-up (14–16). Some studies among sleep apnics reported an elevated incidence of BrCA, PrCA, CRC (17–21) whereas other studies of patients with obstructive sleep apnea (OSA) reported lower risks (22–24). A national matched retrospective cohort among Veterans found that OSA was associated with an increased incidence of BrCA, PrCA, CRC and several other cancers after a median follow-up of 7.4 years (25). It has been suggested that cancer risk increases with OSA severity (17, 26, 27). However, few studies, if any, have examined the role of cumulative sleep-related treatments or duration of diagnosis as proxies for dose-response.

Several studies that assessed sleep either subjectively (sleep quality) or quantitatively (nightly sleep duration) reported no association with cancer incidence (28–31). However, studies among women in Singapore (32) and Ohsaki, Japan (33) both found a modest association between postmenopausal BrCA risk and long sleep duration within a 24-hour period, consistent with a USA study where long sleep (≥9 hours per night) was associated with a modest increase in BrCA risk, although short nightly sleep duration had no association (34). Among men in the Ohsaki cohort, those who reported trouble falling asleep or staying asleep had increased PrCA risks (35) whereas men who slept ≥9 hours per night had lower PrCA risks relative to those with normal sleep (33). These examples indicate that, despite decades of research among millions of patients, there are still inconsistencies and uncertainties among studies examining relationships between sleep disturbances and cancer risk. Recent systematic reviews and meta-analyses addressing this issue highlight heterogeneous sleep assessment methods as one possible explanation, and indicate a need for more studies using clinically diagnosed sleep disorders (8, 16, 17, 31, 36–38).

Racial disparities in sleep disturbances have also been described. African Americans (AAs) have shorter sleep duration, lower sleep efficiency, and unfavorable sleep stages including less slow wave sleep and worse sleep continuity relative to European Americans (EAs) (39, 40). AAs also have an elevated incidence of certain cancers relative to EAs, both in the general (colorectal, gastric, lung, renal, hepatic, prostate) and Veteran (gastric, hepatic, prostate) population (41, 42). However, few studies have examined the extent to which sleep disparities may contribute to racial cancer disparities (40). The VA’s electronic medical record (EMR) system includes data for millions of Veterans and serves as a valuable resource for examining relationships between sleep disorders and cancer incidence.

## Methods

This retrospective cohort study used data from the VA EMR to test the hypothesis that Veterans in the southeastern Veterans Integrated Service Network 7 (VISN-7, includes AL, GA, SC) with a diagnosed sleep disorder had increased cancer risk relative to those without a sleep disorder. Analyses were performed to evaluate whether the duration or severity of the sleep disorder diagnosis enhanced risk, and whether cancer risk was modified by race. Following regulatory approvals, electronic medical records for patients seeking care at least once at a VISN-7 facility between January, 1999 and July, 2010 were retrieved from MedSAS Dataset and Department of Veterans Affairs (VA) Corporate Data Warehouse files. During the study period, the VISN-7 included 9 tertiary care medical centers, 14 community-based outpatient clinics, and 18 primary care clinics serving ~1.3 million Veteran patients, and it was sixth largest among VISNs nationally for percentage of patients in chronic care. Patients younger than 18 years old and those without age information were excluded. Data elements were linked via social security number by a VA data manager, and then scrambled and replaced with a unique patient identification number so that data used by the study investigators were de-identified.

Sleep disorder cases were defined as patients with at least two occurrences of a diagnosis ≥30 days apart based on American Academy of Sleep Medicine International Classification of Sleep Disorder (ICSD) categories (43). The first occurrence of the in- or out-patient diagnosis was used to define date of diagnosis for: sleep disturbances (ICD-9 780.50-59), nonorganic sleep disorders (ICD-9 307.40-49), organic insomnia (ICD-9 327.00-09), organic hypersomnia (ICD-9 327.10-19), organic sleep apnea (ICD-9 327.20-29), and circadian rhythm sleep disorders (ICD-9 327.30-39). Time since diagnosis was calculated by summing the number of months since initial sleep disorder diagnosis and was used in the statistical analyses either as a continuous variable or categorized into three groups based on tertiles defining short, medium and long duration of diagnosis (<1-2.2, 2.3-5.2, 5.3-12.4 years, respectively). Sleep disorder severity was approximated as the cumulative number of treatments by summing sleep-related prescriptions, clinical procedures and surgeries into a single continuous variable. Participants were then grouped into four categories: those without any treatment, and those with few (1 treatment/prescription), moderate (cumulative count: 2-18) or frequent (19 - 1,150) treatments.

Data from the VA Tumor Registry were accessed to identify patients with an incident, primary tumor of the: prostate (ICD-9 185), female breast (ICD-9 174), colorectum (ICD-9 153-4), lung (ICD-9 162), pancreas (ICD-9 157) kidney (ICD-9 189), brain (ICD-9 191), bladder (ICD-9 188), liver (ICD-9 155), ovary (ICD-9 183), esophagus (ICD-9 150), or stomach (ICD-9 151). Patients with a cancer diagnosis prior to the beginning of the study, and those with *in situ* (ICD-9 230-234) and benign (ICD-9 210-229) tumors were excluded. Patients with a rare cancer or an etiology unrelated to the study hypothesis (e.g., infectious) were excluded, which included tumors of the: small intestine (ICD-9 152), skin (ICD-9 172-173), uterus (ICD-9 179 and 182), cervix (ICD-9 180), thyroid (ICD-9 193), lips, oral cavity, pharynx (ICD-9 140-149), or a lymphoma (ICD-9 201). Demographic variables used in the analyses as covariates included: age at cohort entry (18-34 yrs, 35-44 yrs, 45-54 yrs, 55-64 yrs, ≥65 yrs), sex, marital status, and state of residence (AL, GA, SC). Prior to the creating the final analytic data set, Veteran Medicare data were merged by a VA data manager using the social security number to assign race (EA, AA, and Other/Unknown) for each patient based on VA Information Resource Center (VIReC) guidelines (44, 45).

Statistical analyses examining the relationship between sleep disorders and subsequent cancer diagnoses were conducted using extended Cox regression models (SAS 9.3, SAS Institute, Inc, Cary, NC). This method included sleep disorder diagnosis as a time-varying covariate and is applied when the proportional hazards assumption is not met (46, 47). Survival time within the cohort was defined as months from entry until cancer diagnosis or censoring. Patients with only one visit at a VISN-7 facility were censored at the time of entry into the cohort. Hazard ratios with corresponding 95% confidence intervals for each combination of the sleep disorder variable and cancer outcome were computed for both crude and adjusted (age, sex, marital status, state of residence) models with statistical significance set at alpha = 0.05. Initial analyses evaluated all sleep disorders combined and subsequent analyses were performed for the following subgroups: insomnia, sleep apnea, and all other sleep disorders. Analyses of sleep disorder duration were used to assess the latency of tumor development following a sleep disorder diagnosis, and also to address the potential for reverse causality. A time-varying model was implemented where exposure (i.e., sleep disorder diagnosis) was assessed at each failure point in the model, with the amount of time since diagnosis included as a continuous variable in the Cox extended hazard analysis (47). A sleep disorder diagnosis is not always required for a given sleep disorder treatment (i.e., some prescriptions or clinical procedures can be used to treat conditions other than a sleep disorder, or can be used for patients without a sleep disorder diagnosis). Therefore, to prevent the introduction of bias, cumulative sleep-related treatments were computed for all participants regardless of their sleep disorder status, and a Cox extended regression model was used in which the cumulative treatment was multiplied by the total time in the study to account for the lack of proportionality. Additional analyses focused on potential effect modification by race, which was assessed by including an interaction term between race and each sleep disorder variable used in the model, and results were presented as hazard ratios and 95% confidence intervals within each race stratum. In some instances, the stage of disease was missing from the tumor registry. To evaluate the potential introduction of bias, sensitivity analyses included an indicator variable (‘1’ for those without cancer stage and ‘0’ for others) with the Cox extended regression models. Those analyses produced the same conclusions as the results presented below.

## Results

**Figure 1:**
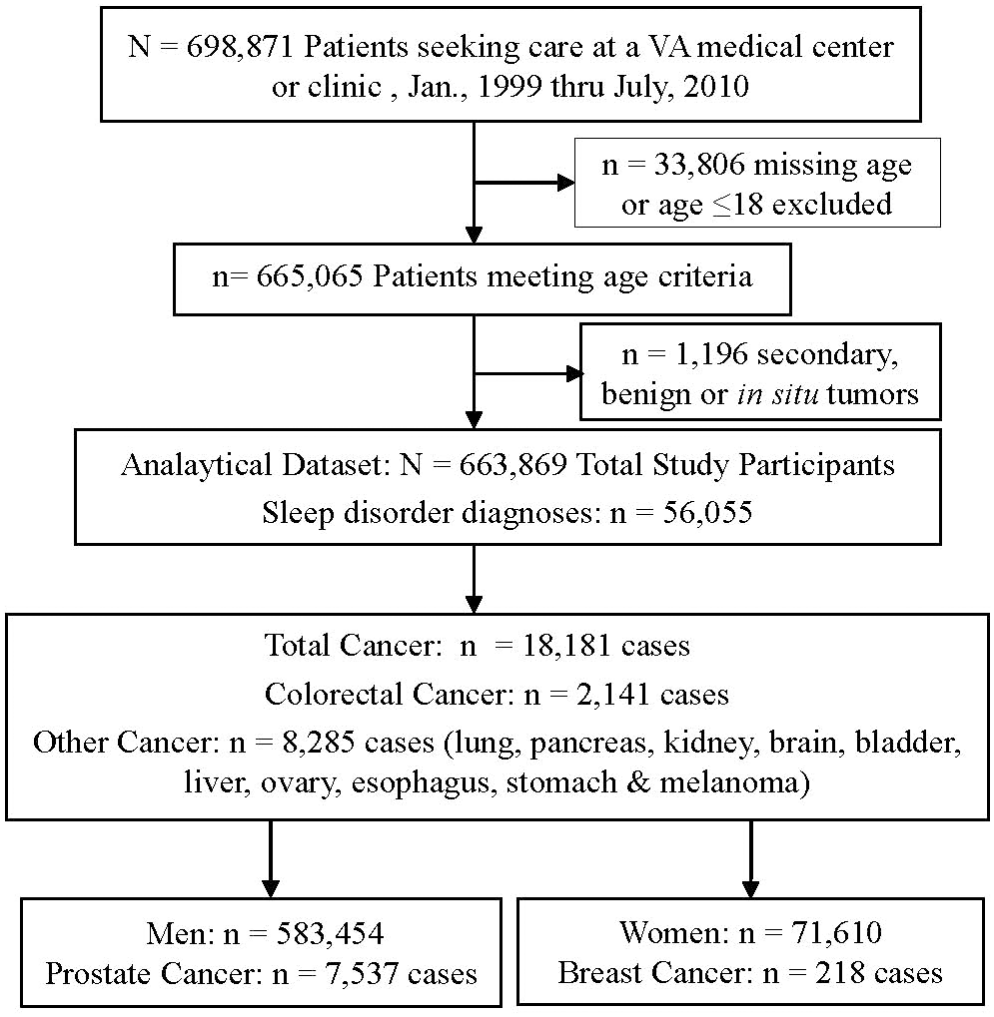
Flow diagram of study population after applying inclusion and exclusion criteria. Patients in the Department of Veterans Affairs (VA) Southeast United States Service Network (VISN-7). Sleep disorder and cancer diagnoses were obtained through the VA’s Corporate Data Warehouse and Tumor Registry, respectively.

The final study population included 663,869 eligible patients after applying inclusion and exclusion criteria (**Figure 1**). At baseline, the study population was 88% male, 45% EA, 26% AA, 29% Other/Unknown, and 67% were ≥45 years old (**Table 1**). There were 56,055 patients with sleep disorders (8.4% of the study population); sleep apnea and insomnia were most common (46% and 40% of all sleep disorders, respectively). Sleep disorder patients were more likely to be African American (30% vs. 26%), male (92% vs. 88%) and divorced (19% vs. 8%) compared to those without a sleep disorder (**Table 1**). The average ( ± SD) duration of a sleep disorder diagnosis was 7.2 ± 3.1 years. Patients with a sleep disorder had an average of 66 ± 98 sleep-related treatments, whereas those without a sleep disorder had an average of 21 ± 56 treatments. The average follow-up time among all patients in the cohort was 10.8 ± 2.5 years. There were 18,181 cancer cases diagnosed during the study period (2.7% of the study population), and the average age at diagnosis was 64 ± 10 years.

**Table 1:** Population characteristics among veterans in the southeast USA (1999-2010, VISN-7).

Characteristic	Total Population (N = 663,869) % (n)	Sleep Disorder (n = 56,055) % (n)	No Sleep Disorder (n = 607,814) % (n)
Age (years)			
18-34	15.4 (102,501)	13.4 (7,524)	15.6 (94,977)
35-44	17.9 (118,952)	19.4 (10,893)	17.8 (108,059)
45-54	26.4 (174,996)	33.4 (18,714)	25.7 (156,282)
55-64	15.7 (104,475)	16.4 (9,164)	15.7 (95,311)
≥65	24.5 (162,945)	17.4 (9,760)	25.2 (153,185)
Race			
African American	26.2 (173,942)	30.1 (16,884)	25.8 (157,058)
European American	44.9 (298,339)	53.8 (30,141)	44.1 (268,198)
Other^1^	28.9 (191,588)	16.1 (9,030)	30.0 (182,558)
Sex			
Male	87.9 (583,454)	92.4 (51,803)	87.5 (531,651)
Female	10.8 (71,610)	6.8 (3,800)	11.2 (67,810)
Unknown	1.3 (8,805)	0.8 (452)	1.4 (8,353)
Marital Status			
Married	51.5 (342,144)	59.2 (33,159)	50.8 (308,955)
Never Married	7.8 (51,846)	6.2 (3,491)	7.9 (48,355)
Divorced	17.8 (118,148)	19.3 (10,827)	7.7 (107,321)
Widowed	6.9 (45,455)	6.1 (3,394)	6.9 (42,061)
Unknown	16.0 (106,306)	9.3 (5,184)	16.6 (101,122)
Cumulative Sleep-Related Treatments^1^			
None (0)	41.2 (273,450)	9.7 (5,456)	44.1 (267,994)
Few (frequency = 1)	7.8 (51,858)	4.6 (2,574)	8.1 (49,284)
Moderate (2 - 18)	25.9 (171,793)	29.3 (16,428)	25.6 (155,365)
Frequent (19 - 1,150)	25.1 (166,768)	56.4 (31,597)	22.2 (135,171)
Cancer Diagnosis			
None	97.3 (645,688)	97.7 (54,784)	97.2 (590,904)
All Cancer	2.7 (18,181)	2.3 (1,271)	2.8 (16,910)
Prostate	1.1 (7,537)	0.92 (515)	1.2 (7,022)
Colorectal	0.32 (2,141)	0.39 (216)	0.32 (1,925)
Female Breast	0.03 (218)	0.03 (15)	0.03 (203)
Other^2^	1.3 (8,285)	0.94 (525)	1.3 (7,760)

^1^ Hispanic, Asian, American Indian, Pacific Islanders, or unknown. ^2^ Includes: lung, pancreas, kidney, brain, bladder, liver, ovary, esophagus, stomach, skin (melanoma). VISN-7, Veterans Integrated Service Network 7 (AL, GA, SC).

Crude and adjusted HRs for cancer incidence in relation to sleep disorder diagnoses are presented in **Table 2**. Patients with any sleep disorder diagnosis, sleep apnea, insomnia, or other types of sleep disorders had increased risks for PrCA, all hypothesized cancers, and other selected cancers (lung, pancreatic, kidney, brain, bladder, liver, ovarian, esophageal, gastric, melanoma). Adjusted HRs for CRC were elevated among those with any sleep disorder or with sleep apnea, whereas there were no statistically significant HRs for female BrCA for any of the sleep disorders (**Table 2**).

**Table 2:** Cancer incidence among veterans with sleep disorders (1999-2010, N=663,869, VISN-7).

Cancer Site	Crude		Adjusted^2^	
	Hazard Ratio	95% CI	Hazard Ratio	95% CI
All Cancer	**Any Sleep Disorder**			
	1.75	(1.66, 1.86)	1.45	(1.37, 1.54)
Prostate	1.73	(1.58, 1.90)	1.50	(1.37, 1.64)
Colorectal	1.52	(1.32, 1.75)	1.34	(1.16, 1.54)
Female Breast	2.87	(1.50, 5.49)	1.69	(0.88, 3.24)
Other^1^	1.75	(1.60, 1.91)	1.45	(1.33, 1.59)
All Cancer	**Insomnia**			
	1.73	(1.58, 1.89)	1.39	(1.27, 1.52)
Prostate	1.64	(1.43, 1.90)	1.39	(1.20, 1.60)
Colorectal	1.46	(1.17, 1.82)	1.22	(0.98, 1.51)
Female Breast	2.98	(1.21, 7.30)	1.78	(0.73, 4.38)
Other^1^	1.84	(1.61, 2.10)	1.46	(1.28, 1.67)
All Cancer	**Sleep Apnea**			
	1.70	(1.55, 1.84)	1.44	(1.32, 1.57)
Prostate	1.72	(1.51, 1.96)	1.52	(1.38, 1.73)
Colorectal	1.48	(1.21, 1.81)	1.38	(1.12, 1.69)
Female Breast	3.31	(1.22, 8.99)	1.90	(0.69, 5.17)
Other^1^	1.59	(1.39, 1.82)	1.37	(1.20, 1.57)
All Cancer	**Other Sleep Disorders** ^3^			
	1.68	(1.46, 1.94)	1.47	(1.28, 1.70)
Prostate	1.69	(1.35, 2.11)	1.54	(1.23, 1.92)
Colorectal	1.53	(1.08, 2.16)	1.41	(0.99, 1.99)
Female Breast^4^	–	–	–	–
Other^1^	1.68	(1.35, 2.09)	1.47	(1.18, 1.83)

^1^ Includes: lung, pancreas, kidney, brain, bladder, liver, ovary, esophagus, stomach, skin (melanoma). ^2^ Adjusted for: age, sex (except for gender specific cancers), marital status, state or residence. CI: confidence interval. ^3^ Includes: hypersomnias, parasomnias, circadian rhythm sleep disorders, movement disorders, arousal disorders. VISN-7, Veterans Integrated Service Network 7 (AL, GA, SC). ^4^ Data too sparse for evaluation.

When the duration of time since diagnosis was evaluated, patients with any sleep disorder had statistically significant increased HRs for all, PrCA, CRC, and for other cancers, with adjusted HRs increasing as the time after initial diagnosis increased (**Table 3**). The results for female BrCA did not indicate a statistically significant increase in cancer risk related to the duration of any sleep disorder (**Table 3**). The results were similar when the data were grouped by those with only insomnia, sleep apnea, or other sleep disorders (results not shown). When cumulative sleep-related treatments were evaluated, statistically significant increases in cancer risk were observed for each type of cancer that was assessed (including BrCA), and HRs increased with an increasing number of prescriptions and clinical procedures (**Table 4**).

**Table 3:** Sleep disorder duration and cancer incidence among veterans (N=663,869, 1999-2010, VISN-7).

Cancer Site	Duration of Sleep Disorder^1^	Crude		Adjusted^2^	
		Hazard Ratio	95% CI	Hazard Ratio	95% CI
All Cancer	Short	1.08	(1.07, 1.10)	1.04	(1.03, 1.06)
	Medium	1.48	(1.39, 1.58)	1.23	(1.16, 1.32)
	Long	2.20	(1.94, 2.50)	1.52	(1.34, 1.73)
Prostate	Short	1.07	(1.05, 1.10)	1.04	(1.02, 1.07)
	Medium	1.43	(1.28, 1.59)	1.24	(1.11, 1.38)
	Long	2.04	(1.65, 2.53)	1.53	(1.24, 1.90)
Colorectal	Short	1.08	(1.06, 1.11)	1.05	(1.03, 1.08)
	Medium	1.48	(1.31, 1.67)	1.29	(1.14, 1.46)
	Long	2.18	(1.70, 2.79)	1.65	(1.29, 2.12)
Female Breast	Short	1.13	(0.96, 1.33)	1.03	(0.87, 1.23)
	Medium	1.86	(0.82, 4.23)	1.17	(0.49, 2.80)
	Long	3.46	(0.67, 17.85)	1.38	(0.24, 7.83)
Other Cancer^3^	Short	1.08	(1.05, 1.10)	1.04	(1.01, 1.06)
	Medium	1.45	(1.30, 1.61)	1.20	(1.07, 1.33)
	Long	2.10	(1.70, 2.59)	1.43	(1.15, 1.77)

^1^ Any sleep disorder of short, medium or long duration since diagnosis (<1-2.2, 2.3-5.2, 5.3-12.4 years, respectively). ^2^ Adjusted for: age, sex (except gender specific cancers), marital status, state of residence. ^3^ Includes lung, pancreas, kidney, brain, bladder, liver, ovary, esophagus, stomach, skin (melanoma). CI, Confidence Interval; VISN-7, Veterans Integrated Service Network 7 (AL, GA, SC).

**Table 4:** Cumulative sleep-related treatments and cancer incidence among veterans (N=663,869, 1999-2010, VISN-7).

Cancer Site	Variable	Cumulative Treatments^1^	Crude		Adjusted^2^	
			Hazard Ratio	95% CI	Hazard Ratio	95% CI
All	Treatment	–	0.96	(0.96, 0.97)	0.96	(0.95, 0.96)
	Treatment*Time	None	1.01	(1.01, 1.01)	1.01	(1.01, 1.01)
		Few	1.22	(1.20, 1.24)	1.26	(1.24, 1.28)
		Moderate	1.49	(1.45, 1.53)	1.60	(1.54, 1.65)
		Frequent	2.22	(2.09, 2.35)	2.55	(2.38, 2.72)
Prostate	Treatment	–	0.95	(0.95, 0.96)	0.94	(0.94, 0.95)
	Treatment*Time	None	1.01	(1.01, 1.01)	1.01	(1.01, 1.01)
		Few	1.31	(1.27, 1.35)	1.35	(1.30, 1.39)
		Moderate	1.71	(1.61, 1.81)	1.81	(1.70, 1.93)
		Frequent	2.91	(2.58, 3.29)	3.28	(2.88, 3.74)
Colorectal	Treatment	–	0.99	(0.98, 0.99)	0.98	(0.97, 0.99)
	Treatment*Time	None	1.00	(1.00, 1.01)	1.00	(1.00, 1.01)
		Few	1.10	(1.06, 1.14)	1.10	(1.06, 1.15)
		Moderate	1.21	(1.11, 1.30)	1.22	(1.12, 1.33)
		Frequent	1.45	(1.24, 1.70)	1.49	(1.25, 1.77)
Female Breast	Treatment	–	0.99	(0.97, 1.00)	0.97	(0.95, 0.99)
	Treatment*Time	None	1.00	(1.00, 1.01)	1.01	(1.00, 1.01)
		Few	1.10	(0.99, 1.23)	1.17	(1.02, 1.34)
		Moderate	1.22	(0.98, 1.51)	1.36	(1.04, 1.78)
		Frequent	1.49	(0.97, 2.29)	1.85	(1.08, 2.18)
Other^3^	Treatment	–	0.97	(0.96, 0.97)	0.96	(0.96, 0.97)
	Treatment*Time	None	1.01	(1.01, 1.01)	1.01	(1.01, 1.01)
		Few	1.20	(1.18, 1.23)	1.24	(1.22, 1.27)
		Moderate	1.45	(1.39, 1.51)	1.54	(1.48, 1.61)
		Frequent	2.09	(1.93, 2.27)	2.38	(2.18, 2.60)

^1^ Cumulative sum of each participant’s sleep-related prescriptions, clinical procedures, and surgeries during the study period; Few (1 treatment or prescription), Moderate (2 - 18), Frequent (19 - 1,150). ^2^ Adjusted for: age, sex (except gender specific cancers), marital status, state of residence. ^3^ Includes: lung, pancreas, kidney, brain, bladder, liver, ovary, esophagus, stomach, skin (melanoma). CI, confidence interval; VISN-7, Veterans Integrated Service Network 7 (AL, GA, SC).

When effect modification by race was examined, few differences were noted among race groups for the main analyses that evaluated the risk of a sleep disorder diagnosis (**Supplementary Table S1A**-**D**), or for analyses that assessed the duration of sleep disorder diagnoses (**Supplementary Table S2**). However, when cumulative sleep-related treatments were stratified by race, cancer risk among AAs was elevated relative to EAs, particularly for all cancer, PrCA, CRC, and Other cancers, and among patients in the ‘Frequent’ cumulative treatment category (**Supplementary Table S3**). For female BrCA, the trends were similar but there were no statistically significant HRs. Results among those in the Other/Unknown race category tended to be intermediate between the AA and EA groups (**Supplementary Table S3**).

## Discussion

In this retrospective cohort study among 663,869 Veterans in the southeastern USA, patients with a sleep disorder diagnosis had increased cancer risks over an average of 11 ± 2.5 years of follow-up. For those with any sleep disorder, the HR was greatest, though imprecise, for BrCA 1.69 (0.88, 3.24), and was lowest for CRC 1.34 (1.16, 1.54). The results are generally consistent with other studies of cancer incidence in populations with sleep disorders, including studies among patients with moderate to severe sleep apnea or insomnia (8, 16, 17, 25, 31, 36, 37). For example, a national study among Veterans with sleep apnea reported an approximate doubling of cancer risk after a median follow-up of 7.4 years (25). To the authors’ knowledge, no other study has assessed cancer risk in relation to the duration of a sleep disorder diagnosis over time, and there were incremental increases in cancer HRs with increasing sleep disorder duration for each type of cancer that was evaluated, suggesting a form of dose-response. In other studies, increased cancer risks were observed in relation to subjective or short-term quantitative sleep disruption measures, but not necessarily in those with a sleep disorder diagnosis. For example, multiple studies have evaluated nightly sleep duration in relation to cancer risk. Short duration sleep (usually defined as ≤6 hours per night) has been associated with PrCA (33), BrCA (48), CRC (49, 50), and total cancer (51), and long nightly sleep has been linked with increased risk of BrCA (34), CRC (50, 52) or other cancers relative to those with normal nightly sleep (usually 7-8 hours per night) (8, 16, 24, 36). The authors also are unaware of other research that evaluated cancer incidence in relation to cumulative sleep-related treatments as a surrogate for disease severity. In a retrospective cohort study (N=33,711), OSA severity and nocturnal hypoxemia were each associated with an increased incidence of cancer of any type (15% and 30% increased risk, respectively) over a median of seven years follow-up (26). In a recent meta-analysis, a monotonic increase in cancer incidence of any type was observed with progressive increases in OSA severity based on apnea-hypopnea indices, with pooled risk estimates ranging from 1.14 (mild), to 1.36 (moderate) and 1.59 (severe) (27). Results in the current study are similar to these observations. Incremental increases in HRs were observed for each of the cancers evaluated as cumulative sleep-related treatments increased relative to those without such treatment. This may also indicate dose-response although other interpretations are possible. A greater number of treatments may suggest an iatrogenic effect of increased cancer risk among those with extended sedative or hypnotic prescription medication use (53), or it may reflect different behaviors, such as heightened health care vigilance or an increased probability of tumor detection by a clinician.

Regardless of the interpretation, cancer risks among those with more sleep-related treatments were greatest among AAs. These results should be interpreted cautiously because evidence for effect modification by race was seen only in analyses of sleep disorder severity, and because all Veterans should have equal access to care. Nonetheless, cancer surveillance data indicate that Veteran AAs have elevated rates of gastric, hepatic, and PrCA incidence relative to EAs (41, 42), which merits further investigation. Racial cancer disparities arise from complex biological, social, and behavioral factors that can include discrimination and stress (39, 40, 54–58). AAs may differ in their endogenous circadian timing relative to EAs (59, 60), and they are more likely than EAs to have poor sleep (39, 40), to participate in shiftwork (61, 62), and to have excessive stress (allostatic overload or ‘weathering’) (54, 63). The autonomic nervous system (ANS) is coupled to sleep and other circadian processes (64–66) as well as the stress response (57, 63, 65, 67, 68). Sleep disturbances elicit stress and chronically can disrupt the ability of the ANS to modulate sympathetic nervous system activity (69, 70). A recent study reported an increased odds of metabolic syndrome, a cancer risk factor (71), among those with both poor sleep and low heart rate variability (HRV, an indicator of dysfunctional autonomic activity), relative to those with normal sleep and HRV (72). This and other evidence indicates that chronic stress leads to a pro-inflammatory state of sympathetic overactivation and ANS dysregulation (73, 74) that can promote tumorigenesis (73–77). These pathophysiological processes may underlie the etiology of racial cancer disparities (57, 58, 63, 64, 68, 74).

This study had some noteworthy strengths and limitations. Strengths include a robust sample size and the use of electronic medical records to ascertain both sleep disorders and cancer diagnoses through the VA’s Corporate Data Warehouse and Tumor Registry, which enhanced validity and provided clinically meaningful exposure and outcome data. The population was restricted to patients in the VA health care system in the southeastern US region (VISN-7), which limits generalizability. The exclusion of tumors with an infectious etiology also limits the generalizability of the results among those with ‘Other Cancers’ since immune dysfunction associated with sleep disturbances and adaptive stress may impact sensitivity to virally mediated cancer progression. Veteran patients are predominantly male (89% in this study population), whereas the distribution of sex in the general population is closer to 50%. For this reason, the statistical power for analyses of BrCA risk may have been insufficient. Veterans who did not utilize a VA medical center, migrated from the study area, or transitioned out of the VA health care system were lost to follow-up, which could have introduced a selection bias. Sleep disorders can occur in heterogeneous populations and prescription use can have multiple indications. The extent to which those factors may influence cancer risk is uncertain. Also, the presence of undiagnosed sleep disorders or other sleep disturbances in the study population may have resulted in exposure misclassification that likely resulted in a bias towards the null. A major study limitation was the inability to control for several important cancer risk factors, such as body mass index, smoking, socioeconomic status, comorbid disease, and family cancer history. Additionally, there was no information on either physical activity or diet, both of which influence carcinogenesis processes including inflammation, and are associated with cancer risk (78, 79). However, a recent study among Veterans reported only modest differences between the crude and adjusted HRs for a cancer diagnosis among those with sleep apnea after adjusting for age, sex, year of cohort entry, smoking, alcohol use, obesity, and comorbid disease (crude HR: 2.16, CI: 2.13-2.20 vs. adjusted HR: 1.97, CI: 1.94-2.00) (25). Among its strengths, this study included innovative analyses of the duration of sleep disorder diagnosis and cumulative sleep-related treatments as potential indicators of dose-response. The increasing HRs with increasing duration or severity of sleep disorder reduced the possibility of confounding but does not rule it out entirely. Similarly, the potential for reverse causality was minimized as the duration of sleep disorder diagnosis or the number of sleep-related treatments increased. The results from this study were consistent when the analyses were performed only among those with sleep apnea, insomnia, or other sleep disorder diagnoses. This suggests that sleep disturbances in general may share a common mechanism of carcinogenesis.

Sleep problems tend to be chronic and persistent (80, 81), and trends in sleep disorders have been increasing both in Veterans and in the general population (3, 4). Night work is an extreme and relevant example of how chronic sleep loss can elicit pathological effects that increase cancer risk (82, 83). The weight of evidence documenting linkages between sleep disruption and cancer incidence has continued to expand (8, 16, 17, 25, 31, 36, 37), although some inconsistencies and uncertainties remain that may be due to differences among study designs, populations studied, or other methodological considerations, particularly sleep assessment validity and representativeness, and uncertainties concerning the nature of the dose-response relationship between sleep disturbances and cancer risk. Results from the current analysis support other evidence suggesting that cancer risks associated with sleep disruption may take up to a decade or more to manifest. This has clinical implications and introduces a window of opportunity for intervention. Concerns over long term sleep medication use (53, 84) suggest a need for alternatives that are safe, effective, low-cost and sustainable. One possibility is supplemental use of melatonin, a hormone derived from the essential amino acid, tryptophan, which facilitates sleep timing and other circadian processes. Multiple studies support its use for insomnia and other sleep problems, particularly in older individuals (85, 86). Melatonin also has established antiproliferative, antioxidant, immune enhancing, and anti-inflammatory properties, and evidence for its effectiveness in treating BrCA, PrCA, CRC and other cancers is promising (87, 88). However, its role in cancer prevention has not been thoroughly studied. Another promising class of compounds are used for insomnia treatment by acting upon the orexin (hypocretin) receptor system that modulates sleep/wakefulness (89). These agents selectively induce apoptosis in several tumor types and thus have promising therapeutic potential (8). Other alternative sleep disorder treatments include interventions that act upon ANS activity, such as HRV biofeedback, which is effective at improving sleep and reducing inflammation using a nonpharmacological, mechanism-driven technique (resonance frequency breathing) that can reduce sympathetic overactivation and promote ANS homeostasis (73, 74, 90, 91). Additional research is needed to further optimize the timing, frequency and intensity of these and other innovative sleep promoting, cancer prevention strategies in order to fully characterize their effectiveness and long term benefits.

In summary, the results of this study are consistent with other data indicating a link between sleep perturbations and cancer risk. Sleep disorder diagnoses and clinical treatments were examined rather than short-term measures of nightly sleep or self-reported sleep quality. Patients with a longer duration or greater severity of sleep disorder diagnosis had the greatest risks. The findings suggest that optimal sleep and appropriate sleep disorder management may represent modifiable risk factors for facilitating cancer prevention. Carefully controlled clinical trials are needed to more rigorously evaluate these possibilities.

## Data availability statement

The data analyzed in this study was obtained from USA Department of Veterans Affairs (VA) Corporate Data Warehouse and MedSAS data files. Requests to access the datasets should be directed to the VA Informatics and Computing Infrastructure (VINCI) office (VINCI@VA.GOV).

## Ethics statement

The study was conducted in accordance with applicable laws and regulations governing use of secondary data in human research. Data were coded to protect the identity of study subjects. This study was reviewed and approved by the Institutional Review Boards of the Columbia Veterans Affairs Health Care System (formerly WJB Dorn Department of Veterans Affairs Medical Center) and the University of South Carolina, Columbia, SC.

## Author contributions

JBB: Conceptualization, Methodology, Funding acquisition, Project administration, Investigation, Data curation, Software, Formal Analysis, Validation, Writing – original draft, Writing – review & editing. AFD: Conceptualization, Methodology, Project administration, Investigation, Data curation, Software, Formal Analysis, Validation, Writing – original draft, Writing – review & editing. HZ: Conceptualization, Methodology, Investigation, Data curation, Software, Formal Analysis, Validation, Writing – review & editing. ACM: Conceptualization, Methodology, Investigation, Data curation, Software, Formal Analysis, Validation, Writing – review & editing. MAR: Investigation, Data curation, Software, Formal Analysis, Validation, Writing – review & editing. AM: Writing – original draft, Writing – review & editing. SAA: Conceptualization, Methodology, Writing – review & editing. JRH: Conceptualization, Methodology, Writing – review & editing.

## Supplementary material

Supplementary Table 1a All Sleep Disorder Diagnoses and Cancer Incidence Stratified by Race, Veterans in the Southeast USA (1999-2010, VISN-7).

Supplementary Table 1b Insomnia Diagnoses and Cancer Incidence Stratified by Race, Veterans in the Southeast USA (1999-2010, VISN-7).

Supplementary Table 1c Sleep Apnea Diagnosis and Cancer Incidence stratified by Race, Veterans in the Southeast USA (1999-2010, VISN-7).

Supplementary Table 1d Other Sleep Disorder Diagnoses and Cancer Incidence stratified by Race, Veterans in the Southeast USA (1999-2010, VISN-7).

Supplementary Table 2 Duration of Sleep Disorder Diagnosis and Cancer Incidence Stratified by Race, Veterans in the SE USA (VISN-7, 1999-2010).

Supplementary Table 3 Cumulative Sleep-Related Treatments and Cancer Incidence Among Veterans, Stratified by Race (VISN-7, 1999-2010).
