# Supplementary material for: Sleep disorders and cancer incidence: examining duration and severity of diagnosis among veterans
Source: Front Oncol. 2024 Feb 26;14:1336487. doi: 10.3389/fonc.2024.1336487 (PMC10927008; doi:10.3389/fonc.2024.1336487)
Supplement: Supplementary Table 1a — All Sleep Disorder Diagnoses and Cancer Incidence Stratified by Race, Veterans in the Southeast USA (1999-2010, VISN-7). [file Table_1.docx]

| **Table S.1a. All Sleep Disorder Diagnoses and Cancer Incidence Stratified by Race,**  **Veterans in the Southeast USA (1999-2010, VISN-7)** | | | | | |
| --- | --- | --- | --- | --- | --- |
| **Cancer Site** | **Sleep Disorder Type** | **Race** | **N** | **Adjusted**  **Hazard Ratio**^2^ | **95% CI** |
| All | All | African American | 173,942 | 1.28 | (1.16, 1.41) |
|  |  | European American | 298,339 | 1.37 | (1.27, 1.48) |
|  |  | Unknown/Other | 191,588 | 1.76 | (1.41, 2.20) |
| Prostate | All | African American | 150,732 | 1.46 | (1.29, 1.67) |
|  |  | European American | 279,199 | 1.33 | (1.15, 1.53) |
|  |  | Unknown/Other | 153,523 | 2.03 | (1.52, 2.73) |
| Colorectal | All | African American | 173,942 | 1.04 | (0.81, 1.33) |
|  |  | European American | 298,339 | 1.35 | (1.23, 1.61) |
|  |  | Unknown/Other | 191,588 | 1.21 | (0.59, 2.48) |
| Female Breast | All | African American | 20,994 | 1.03 | (0.37, 2.86) |
|  |  | European American | 15,610 | 1.31 | (0.47, 3.67) |
|  |  | Unknown/Other | 35,006 | 7.75 | (1.64, 36.56) |
| Other^1^ | All | African American | 173,942 | 1.15 | (0.96, 1.37) |
|  |  | European American | 298,339 | 1.39 | (1.25, 1.54) |
|  |  | Unknown/Other | 191,588 | 1.52 | (1.01, 2.28) |
| ^1^ Includes: lung, pancreas, kidney, brain, bladder, liver, ovary, esophagus, stomach, skin (melanoma). ^2^ Adjusted for: age, sex (except for gender specific cancers), marital status, state or residence. CI: confidence interval. VISN-7: Veterans Integrated Service Network 7 (AL, GA, SC). | | | | | |

| **Table S.1b. Insomnia Diagnoses and Cancer Incidence Stratified by Race,**  **Veterans in the Southeast USA (1999-2010, VISN-7)** | | | | | |
| --- | --- | --- | --- | --- | --- |
| **Cancer Site** | **Sleep Disorder Type** | **Race** | **N** | **Adjusted**  **Hazard Ratio**^2^ | **95% CI** |
| All | Insomnias | African American | 173,942 | 1.33 | (1.15, 1.53) |
|  |  | European American | 298,339 | 1.27 | (1.13, 1.43) |
|  |  | Unknown/Other | 191,588 | 1.65 | (1.18, 2.30) |
| Prostate | Insomnias | African American | 150,732 | 1.44 | (1.18, 1.76) |
|  |  | European American | 279,199 | 1.12 | (0.88, 1.42) |
|  |  | Unknown/Other | 153,523 | 2.06 | (1.36, 3.14) |
| Colorectal | Insomnias | African American | 173,942 | 1.21 | (0.85, 1.72) |
|  |  | European American | 298,339 | 1.14 | (0.86, 1.51) |
|  |  | Unknown/Other | 191,588 | 0.34 | (0.05, 2.41) |
| Female Breast | Insomnias | African American | 20,994 | 0.57 | (0.08, 4.13) |
|  |  | European American | 15,610 | 2.01 | (0.62, 6.48) |
|  |  | Unknown/Other | 35,006 | 6.95 | (0.87, 55.4) |
| Other^1^ | Insomnias | African American | 173,942 | 1.24 | (0.95, 1.62) |
|  |  | European American | 298,339 | 1.40 | (1.19, 1.64) |
|  |  | Unknown/Other | 191,588 | 1.47 | (0.81, 2.68) |
| ^1^ Includes: lung, pancreas, kidney, brain, bladder, liver, ovary, esophagus, stomach, skin (melanoma). ^2^ Adjusted for: age, sex (except for gender specific cancers), marital status, state or residence. CI: confidence interval. VISN-7: Veterans Integrated Service Network 7 (AL, GA, SC). | | | | | |

| **Table S.1c. Sleep Apnea Diagnoses and Cancer Incidence stratified by Race,**  **Veterans in the Southeast USA (1999-2010, VISN-7)** | | | | | |
| --- | --- | --- | --- | --- | --- |
| **Cancer Site** | **Sleep Disorder Type** | **Race** | **N** | **Adjusted**  **Hazard Ratio**^2^ | **95% CI** |
| All | Apneas | African American | 173,942 | 1.16 | (1.00, 1.35) |
|  |  | European American | 298,339 | 1.41 | (1.27, 1.57) |
|  |  | Unknown/Other | 191,588 | 1.80 | (1.29, 2.51) |
| Prostate | Apneas | African American | 150,732 | 1.40 | (1.16, 1.69) |
|  |  | European American | 279,199 | 1.45 | (1.19, 1.77) |
|  |  | Unknown/Other | 153,523 | 1.97 | (1.26, 3.08) |
| Colorectal | Apneas | African American | 173,942 | 0.85 | (0.57, 1.26) |
|  |  | European American | 298,339 | 1.50 | (1.17, 1.91) |
|  |  | Unknown/Other | 191,588 | 1.41 | (0.52, 3.84) |
| Female Breast | Apneas | African American | 20,994 | 1.41 | (0.34, 5.78) |
|  |  | European American | 15,610 | 0.98 | (0.14, 7.14) |
|  |  | Unknown/Other | 35,006 | 10.42 | (1.29, 84.1) |
| Other^1^ | Apneas | African American | 173,942 | 0.96 | (0.72, 1.29) |
|  |  | European American | 298,339 | 1.34 | (1.14, 1.57) |
|  |  | Unknown/Other | 191,588 | 1.62 | (0.89, 2.95) |
| ^1^ Includes: lung, pancreas, kidney, brain, bladder, liver, ovary, esophagus, stomach, skin (melanoma). ^2^ Adjusted for: age, sex (except for gender specific cancers), marital status, state or residence. CI: confidence interval. VISN-7: Veterans Integrated Service Network 7 (AL, GA, SC). | | | | | |

| **Table S.1d. Other Sleep Disorder Diagnoses and Cancer Incidence stratified by Race,**  **Veterans in the Southeast USA (1999-2010, VISN-7)** | | | | | |
| --- | --- | --- | --- | --- | --- |
| **Cancer Site** | **Sleep Disorder Type** | **Race** | **N** | **Adjusted**  **Hazard Ratio**^3^ | **95% CI** |
| All | Other^2^ | African American | 173,942 | 1.39 | (1.11, 1.74) |
|  |  | European American | 298,339 | 1.33 | (1.09, 1.61) |
|  |  | Unknown/Other | 191,588 | 1.73 | (0.98, 3.05) |
| Prostate | Other^2^ | African American | 150,732 | 1.51 | (1.11, 2.06) |
|  |  | European American | 279,199 | 1.34 | (0.97, 1.95) |
|  |  | Unknown/Other | 153,523 | 1.70 | (0.76, 3.80) |
| Colorectal | Other^2^ | African American | 173,942 | 1.11 | (0.61, 2.02) |
|  |  | European American | 298,339 | 1.27 | (0.81, 2.01) |
|  |  | Unknown/Other | 191,588 | 3.28 | (1.04, 10.3) |
| Other^1^ | Other^2^ | African American | 173,942 | 1.36 | (0.90, 2.06) |
|  |  | European American | 298,339 | 1.34 | (1.03, 1.75) |
|  |  | Unknown/Other | 191,588 | 1.26 | (0.41, 3.93) |
| ^1^ Includes: lung, pancreatic, kidney, brain, bladder, liver, ovarian, esophageal, gastric cancers, and melanoma. Data too sparse for evaluation of female breast cancer. ^2^ Includes: hypersomnia, parasomnia, circadian rhythm, movement disorders, and arousal disorders. ^3^ Adjusted for: marital status, state or residence, sex (except for gender specific cancers) and age. CI: Confidence Interval. | | | | | |
