# Supplementary material for: Sleep disorders and cancer incidence: examining duration and severity of diagnosis among veterans
Source: Front Oncol. 2024 Feb 26;14:1336487. doi: 10.3389/fonc.2024.1336487 (PMC10927008; doi:10.3389/fonc.2024.1336487)
Supplement: Supplementary Table 2 — Duration of Sleep Disorder Diagnosis and Cancer Incidence Stratified by Race, Veterans in the SE USA (VISN-7, 1999-2010). [file Table_2.docx]

| **Table S.2. Duration of Sleep Disorder Diagnosis and Cancer Incidence Stratified by Race, Veterans in the SE USA (VISN-7, 1999-2010)** | | | | |
| --- | --- | --- | --- | --- |
| **Cancer Site** | **Duration of Sleep Disorder (years)**^1^ | **Race** | **Adjusted**  **Hazard Ratio**^2^ | **95% CI** |
| All | Short | European American | 1.00 | (1.00, 1.01) |
|  |  | African American | 1.00 | (1.00, 1.01) |
|  |  | Other/Unknown | 1.00 | (1.00, 1.01) |
|  | Medium | European American | 1.17 | (1.08, 1.28) |
|  |  | African American | 1.12 | (1.01, 1.25) |
|  |  | Other/Unknown | 1.30 | (0.99, 1.71) |
|  | Long | European American | 1.38 | (1.16, 1.64) |
|  |  | African American | 1.26 | (1.02, 1.56) |
|  |  | Other/Unknown | 1.69 | (0.98, 2.93) |
| Prostate | Short | European American | 1.00 | (0.99, 1.01) |
|  |  | African American | 1.00 | (1.00, 1.01) |
|  |  | Other/Unknown | 1.01 | (1.00, 1.01) |
|  | Medium | European American | 1.11 | (0.93, 1.32) |
|  |  | African American | 1.19 | (1.02, 1.38) |
|  |  | Other/Unknown | 1.66 | (1.18, 2.33) |
|  | Long | European American | 1.23 | (0.87, 1.75) |
|  |  | African American | 1.41 | (1.05, 1.90) |
|  |  | Other/Unknown | 2.76 | (1.40, 5.45) |
| Colorectal | Short | European American | 1.00 | (1.00, 1.01) |
|  |  | African American | 1.00 | (0.99, 1.01) |
|  |  | Other/Unknown | 0.99 | (0.99, 1.01) |
|  | Medium | European American | 1.27 | (1.09, 1.49) |
|  |  | African American | 1.14 | (0.91, 1.41) |
|  |  | Other/Unknown | 0.92 | (0.42, 2.02) |
|  | Long | European American | 1.62 | (1.18, 2.21) |
|  |  | African American | 1.29 | (0.83, 1.99) |
|  |  | Other/Unknown | 0.85 | (0.18, 4.10) |
| Female Breast | Short | European American | 0.99 | (0.96, 1.02) |
|  |  | African American | 1.00 | (0.98, 1.02) |
|  |  | Other/Unknown | 1.02 | (0.98, 1.06) |
|  | Medium | European American | 0.52 | (0.07, 3.85) |
|  |  | African American | 1.18 | (0.39, 3.59) |
|  |  | Other/Unknown | 3.25 | (0.38, 27.8) |
|  | Long | European American | 0.27 | (0.01, 14.8) |
|  |  | African American | 1.40 | (0.15, 12.9) |
|  |  | Other/Unknown | 10.58 | (0.15, 770.7) |
| ^1^ Time since sleep disorder diagnosis grouped as short (<1-2.2 yrs), medium (2.3-5.2 yrs), and long (5.3-12.4 years). ^2^ Adjusted for: age, sex (except gender specific cancers), marital status, state of residence. CI: Confidence Interval. VISN-7: Veterans Integrated Service Network 7 (AL, GA, SC). | | | | |

| **Table S.2 (continued). Duration of Sleep Disorder Diagnosis and Cancer Incidence Stratified by Race, Veterans in the SE USA**  **(VISN-7, 1999-2010)** | | | | |
| --- | --- | --- | --- | --- |
| **Cancer Site** | **Duration of Sleep Disorder (years)**^1^ | **Race** | **Adjusted**  **Hazard Ratio**^2^ | **95% CI** |
| Other^3^ | Short | European American | 1.00 | (1.00, 1.01) |
|  |  | African American | 1.00 | (0.99, 1.00) |
|  |  | Other/Unknown | 0.99 | (0.99, 1.01) |
|  | Medium | European American | 1.15 | (1.02, 1.46) |
|  |  | African American | 1.01 | (0.82, 1.25) |
|  |  | Other/Unknown | 0.93 | (0.51, 1.70) |
|  | Long | European American | 1.33 | (1.03, 1.72) |
|  |  | African American | 1.02 | (0.67, 1.57) |
|  |  | Other/Unknown | 0.87 | (0.26, 2.89) |
| ^1^ Time since sleep disorder diagnosis grouped as short (<1-2.2 yrs), medium (2.3-5.2 yrs), and long (5.3-12.4 years). ^2^ Adjusted for: age, sex (except gender specific cancers), marital status, state of residence. ^3^ Includes lung, pancreas, kidney, brain, bladder, liver, ovary, esophagus, stomach, skin (melanoma). CI: Confidence Interval. VISN-7: Veterans Integrated Service Network 7 (AL, GA, SC). | | | | |
