# Supplementary material for: Sleep disorders and cancer incidence: examining duration and severity of diagnosis among veterans
Source: Front Oncol. 2024 Feb 26;14:1336487. doi: 10.3389/fonc.2024.1336487 (PMC10927008; doi:10.3389/fonc.2024.1336487)
Supplement: Supplementary Table 3 — Cumulative Sleep-Related Treatments and Cancer Incidence Among Veterans, Stratified by Race (VISN-7, 1999-2010). [file Table_3.docx]

| **Table S.3.**  **Cumulative Sleep-Related Treatments and Cancer Incidence Among Veterans,**  **Stratified by Race (1999-2010, VISN-7)** | | | | | | |
| --- | --- | --- | --- | --- | --- | --- |
| **Cancer Site** | **Sleep Disorder Treatment Variable** | **Cumulative Treatment**^1^ | **Race** | **N**^2^ | **Adjusted**  **Hazard Ratio**^3^ | **95% CI** |
| All | Treatment | -- | European American | 298,339 | 0.96 | (0.95, 0.96) |
|  |  |  | African American | 173,942 | 0.93 | (0.92, 0.94) |
|  |  |  | Other/Unknown | 191,588 | 0.94 | (0.92, 0.96) |
|  | Treatment*Time | None | European American |  | 1.01 | (1.01, 1.01) |
|  |  |  | African American |  | 1.02 | (1.01, 1.02) |
|  |  |  | Other/Unknown |  | 1.01 | (1.01, 1.02) |
|  |  | Few | European American |  | 1.25 | (1.22, 1.27) |
|  |  |  | African American |  | 1.44 | (1.39, 1.50) |
|  |  |  | Other/Unknown |  | 1.41 | (1.28, 1.55) |
|  |  | Moderate | European American |  | 1.56 | (1.50, 1.62) |
|  |  |  | African American |  | 2.08 | (1.92, 2.25) |
|  |  |  | Other/Unknown |  | 1.98 | (1.64, 2.39) |
|  |  | Frequent | European American |  | 2.42 | (2.24, 2.62) |
|  |  |  | African American |  | 4.32 | (3.68, 5.07) |
|  |  |  | Other/Unknown |  | 3.92 | (2.70, 5.70) |
| Prostate | Treatment | -- | European American | 279,199 | 0.95 | (0.94, 0.96) |
|  |  |  | African American | 150,732 | 0.92 | (0.91, 0.93) |
|  |  |  | Other/Unknown | 153,523 | 0.93 | (0.91, 0.96) |
|  | Treatment*Time | None | European American |  | 1.01 | (1.01, 1.01) |
|  |  |  | African American |  | 1.02 | (1.02, 1.02) |
|  |  |  | Other/Unknown |  | 1.02 | (1.01, 1.02) |
|  |  | Few | European American |  | 1.31 | (1.26, 1.37) |
|  |  |  | African American |  | 1.54 | (1.44, 1.64) |
|  |  |  | Other/Unknown |  | 1.46 | (1.27, 1.68) |
|  |  | Moderate | European American |  | 1.72 | (1.58, 1.87) |
|  |  |  | African American |  | 2.36 | (2.07, 2.69) |
|  |  |  | Other/Unknown |  | 2.13 | (1.61, 2.82) |
|  |  | Frequent | European American |  | 2.96 | (2.51, 3.49) |
|  |  |  | African American |  | 5.56 | (4.28, 7.22) |
|  |  |  | Other/Unknown |  | 4.54 | (2.61, 7.93) |
| ^1^ Cumulative sum of each participant’s sleep-related prescriptions, clinical procedures, and surgeries during the study period; Few (1 treatment or prescription), Moderate (2 - 18), Frequent (19 - 1,150). ^2^ N presented for the main treatment effect. ^3^ Adjusted for: age, sex (except gender specific cancers), marital status, state of residence.  CI: confidence interval. VISN-7: Veterans Integrated Service Network 7 (AL, GA, SC). | | | | | | |

| **Table S.3 (continued).**  **Cumulative Sleep-Related Treatments and Cancer Incidence Among Veterans, Stratified by Race (1999-2010, VISN-7)** | | | | | | |
| --- | --- | --- | --- | --- | --- | --- |
| **Cancer Site** | **Sleep Disorder Treatment Variable** | **Cumulative Treatment**^1^ | **Race** | **N**^2^ | **Adjusted**  **Hazard Ratio**^3^ | **95% CI** |
| Colorectal | Treatment | -- | European American | 298,339 | 0.99 | (0.98, 0.99) |
|  |  |  | African American | 173,942 | 0.97 | (0.95, 0.99) |
|  |  |  | Other/Unknown | 191,588 | 0.98 | (0.93, 1.03) |
|  | Treatment*Time | None | European American |  | 1.00 | (1.00, 1.01) |
|  |  |  | African American |  | 1.01 | (1.00, 1.01) |
|  |  |  | Other/Unknown |  | 1.01 | (0.99, 1.02) |
|  |  | Few | European American |  | 1.09 | (1.04, 1.15) |
|  |  |  | African American |  | 1.20 | (1.08, 1.34) |
|  |  |  | Other/Unknown |  | 1.13 | (0.86, 1.49) |
|  |  | Moderate | European American |  | 1.19 | (1.07, 1.32) |
|  |  |  | African American |  | 1.44 | (1.16, 1.78) |
|  |  |  | Other/Unknown |  | 1.28 | (0.74, 2.22) |
|  |  | Frequent | European American |  | 1.41 | (1.15, 1.73) |
|  |  |  | African American |  | 2.07 | (1.34, 3.18) |
|  |  |  | Other/Unknown |  | 1.65 | (0.55, 4.92) |
| Female Breast | Treatment | -- | European American | 15,610 | 0.96 | (0.93, 1.00) |
|  |  |  | African American | 20,994 | 0.96 | (0.92, 1.00) |
|  |  |  | Other/Unknown | 35,006 | 0.92 | (0.65, 1.29) |
|  | Treatment*Time | None | European American |  | 1.01 | (0.99, 1.02) |
|  |  |  | African American |  | 1.01 | (1.00, 1.02) |
|  |  |  | Other/Unknown |  | 1.02 | (0.95, 1.10) |
|  |  | Few | European American |  | 1.21 | (0.98, 1.48) |
|  |  |  | African American |  | 1.24 | (0.99, 1.56) |
|  |  |  | Other/Unknown |  | 1.58 | (0.26, 9.67) |
|  |  | Moderate | European American |  | 1.46 | (0.97, 2.20) |
|  |  |  | African American |  | 1.55 | (0.99, 2.42) |
|  |  |  | Other/Unknown |  | 2.50 | (0.07, 93.5) |
|  |  | Frequent | European American |  | 2.13 | (0.94, 4.83) |
|  |  |  | African American |  | 2.40 | (0.98, 5.85) |
|  |  |  | Other/Unknown |  | 6.23 | (0.01, 8,740) |
| ^1^ Cumulative sum of each participant’s sleep-related prescriptions, clinical procedures, and surgeries during the study period; Few (1 treatment or prescription), Moderate (2 - 18), Frequent (19 - 1,150). ^2^ N presented for the main treatment effect. ^3^ Adjusted for: age, sex (except gender specific cancers), marital status, state of residence.  CI: confidence interval. VISN-7: Veterans Integrated Service Network 7 (AL, GA, SC). | | | | | | |

| **Table S.3 (continued).**  **Cumulative Sleep-Related Treatments and Cancer Incidence Among Veterans, Stratified by Race (1999-2010, VISN-7)** | | | | | | |
| --- | --- | --- | --- | --- | --- | --- |
| **Cancer Site** | **Sleep Disorder Treatment Variable** | **Cumulative Treatment**^1^ | **Race** | **N**^2^ | **Adjusted**  **Hazard Ratio**^3^ | **95% CI** |
| Other^4^ | Treatment | -- | European American | 298,339 | 0.96 | (0.95, 0.97) |
|  |  |  | African American | 173,942 | 0.94 | (0.93, 0.95) |
|  |  |  | Other/Unknown | 191,588 | 0.94 | (0.91, 0.96) |
|  | Treatment*Time | None | European American |  | 1.01 | (1.00, 1.01) |
|  |  |  | African American |  | 1.01 | (1.01, 1.02) |
|  |  |  | Other/Unknown |  | 1.01 | (1.01, 1.02) |
|  |  | Few | European American |  | 1.24 | (1.21, 1.28) |
|  |  |  | African American |  | 1.41 | (1.33, 1.50) |
|  |  |  | Other/Unknown |  | 1.43 | (1.22, 1.67) |
|  |  | Moderate | European American |  | 1.54 | (1.47, 1.63) |
|  |  |  | African American |  | 1.99 | (1.76, 2.26) |
|  |  |  | Other/Unknown |  | 2.03 | (1.49, 2.77) |
|  |  | Frequent | European American |  | 2.39 | (2.15, 2.64) |
|  |  |  | African American |  | 3.96 | (3.09, 5.09) |
|  |  |  | Other/Unknown |  | 4.14 | (2.22, 7.69) |
| ^1^ Cumulative sum of each participant’s sleep-related prescriptions, clinical procedures, and surgeries during the study period; Few (1 treatment or prescription), Moderate (2 - 18), Frequent (19 - 1,150). ^2^ N presented for the main treatment effect. ^3^ Adjusted for: age, sex (except gender specific cancers), marital status, state of residence. ^4^ Includes: lung, pancreatic, kidney, brain, bladder, liver, ovarian, esophageal, gastric cancers, and melanoma. CI: confidence interval. VISN-7: Veterans Integrated Service Network 7 (AL, GA, SC). | | | | | | |
